# The Epidemiology and Economic Burden of *Clostridium difficile* Infection in Korea

**DOI:** 10.1155/2015/510386

**Published:** 2015-03-02

**Authors:** Hyung-Yun Choi, So-Youn Park, Young-Ae Kim, Tai-Young Yoon, Joong-Myung Choi, Bong-Keun Choe, So-Hee Ahn, Seok-Jun Yoon, Ye-Rin Lee, In-Hwan Oh

**Affiliations:** ^1^Department of Preventive Medicine, School of Medicine, Kyung Hee University, Seoul 130-701, Republic of Korea; ^2^Department of Medical Education and Medical Humanities, School of Medicine, Kyung Hee University, Seoul 130-701, Republic of Korea; ^3^Cancer Policy Branch, National Cancer Control Institute, National Cancer Center, Goyang 410-769, Republic of Korea; ^4^Department of Preventive Medicine, College of Medicine, Korea University, Seoul 136-701, Republic of Korea

## Abstract

The prevalence of *Clostridium difficile* infection and the associated burden have recently increased in many countries. While the main risk factors for *C. difficile* infection include old age and antibiotic use, the prevalence of this infection is increasing in low-risk groups. These trends highlight the need for research on *C. difficile* infection. This study pointed out the prevalence and economic burden of *C. difficile* infection and uses the representative national data which is primarily from the database of the Korean Health Insurance Review and Assessment Service, for 2008–2011. The annual economic cost was measured using a prevalence approach, which sums the costs incurred to treat *C. difficile* infection. *C. difficile* infection prevalence was estimated to have increased from 1.43 per 100,000 in 2008 to 5.06 per 100,000 in 2011. Moreover, mortality increased from 69 cases in 2008 to 172 in 2011. The economic cost increased concurrently, from $2.4 million in 2008 to $7.6 million, $10.5 million, and $15.8 million in 2009, 2010, and 2011, respectively. The increasing economic burden of *C. difficile* infection over the course of the study period emphasizes the need for intervention to minimize the burden of a preventable illness like *C. difficile* infection.

## 1. Introduction

*Clostridium difficile *is spore-forming, Gram-positive anaerobic bacteria that produces enterotoxin A (TcdA) and cytotoxin B (TcdB), which cause diarrhea [1–3]. In addition to diarrhea,* C*.* difficile* infection (CDI) can cause colitis and life-threatening sepsis [2, 3]. These disease incidences are increasing worldwide. In Korea, there are some studies supporting this fact. In a single-hospital study, the incidence of CDI increased 6-fold during the period of 1998–2007, from 1.9 inpatients per 10,000 persons in 1998 to 8.82 in 2006-2007 [4]. In another study of 17 hospitals for the period of 2004–2008, the number of adult (over 19 years) inpatients with CDI per 1,000 persons increased from 1.7 in 2004 to 2.7 in 2008 [5].

The main risk factors for CDI include old age, antibiotic use, and long-term hospital stays. Other known CDI risk factors are intensive care unit (ICU) admission, mechanical ventilation, parenteral nutrition, hemodialysis, and gastrointestinal disorders [6–14]. The most important risk factor for CDI is antibiotic use, especially the use of clindamycin, cephalosporins, penicillins, and fluoroquinolones [15–17].

Some cases without such risk factors have been reported, and the prevalence and severity of CDI in low-risk groups are increasing in many countries, including the USA, Canada, and Japan [18, 19]. These changes could be due to alterations in host susceptibility, antibiotic therapy, and hospital infection management [10, 20]. The epidemiology of CDI has also dramatically changed. A previously unknown restriction enzyme analysis type of* C. difficile*, known as BI/North American pulsed-field gel electrophoresis type 1 (BI/NAP1), is especially resistant to fluoroquinolones and possesses an additional gene encoding for the binary toxin [10]. To minimize exposure to* C. difficile* during hospitalization, infection controls such as contact precautions and hand hygiene are important and could decrease the costs associated with infection [21].

The importance of CDI is emphasized by changes in the prevalence pattern of the disease. Although much research is being conducted on the subject [18, 19, 22], few studies have examined the epidemiology and economic burden of CDI. Given the increasing prevalence of CDI and associated mortality in Korea, this study determined the prevalence and economic burden of CDI using Korean representative national data.

## 2. Materials and Methods

This study estimated socioeconomic burden using a prevalence-based approach and estimated the costs arising from CDI patients and mortality during the period of 2008–2011 in Korea.

Data on the number of patients and associated health insurance costs were obtained from claims data of the Health Insurance Review and Assessment Service [23]. Cases in which the principal diagnosis was the International Classification of Diseases (ICD) diagnostic code A04.7 on the insurance claim during the period 2008–2011 were identified, and the costs were calculated for each year. The Korean National Health Insurance system covers all residents of Korea, and several studies have attempted to estimate prevalence using the National Health Insurance data [24–26]. A death was defined as a case given the same code (A04.7) as the cause of death in National Statistical Office data for the corresponding year [27]. Previous studies of CDI prevalence and mortality defined the disease based on ICD code A04.7, which corresponds to enterocolitis caused by* C. difficile* and was considered to include deaths related to CDI [28, 29].

Annual prevalence and mortality were compared based on calculations from the National Statistical Office data on the projected population [27]. In addition, the economic burden for each of three age groups (0–19, 20–64, and ≥65) was estimated. The estimated total and CDI-associated socioeconomic costs were calculated by summing the direct and indirect costs [25]. All estimated costs are presented in U.S. dollars (USD), with the conversion rate of 1,045 won/USD, based on the exchange rate on January 1, 2014. The calculation method was as follows [25]:

(1) Total  costs=Direct  costs+Indirect  costs,Direct  costs=Inpatient  care  costs+Outpatient  costsDirect  costs=+Medication  costs+Transportation  costs,Indirect  costs=Productivity  loss  (morbidity)Indirect  costs=+Productivity  loss  (premature  mortality)Indirect  costs=+Caregiver  costs.

Direct costs included the direct medical costs, including expenses arising from inpatient and outpatient care and drug costs, and direct nonmedical costs, such as costs associated with transportation to medical services and facilities. Here, direct medical costs included both insured and uninsured medical expenses. Insured medical expenses included the amount paid by health insurance and the patients' copayments, while uninsured medical expenses (those not covered by the Korean National Health Insurance system) were estimated based on the percentage of uninsured expenses in data from a survey on medical charges [30]. Transportation costs were estimated from Korea Health Panel Survey data of transportation expenses paid by patients who underwent medical care for a digestive disease [31].

Indirect costs included CDI-caused productivity losses, costs caused by premature mortality, and caregivers' costs. CDI-associated productivity losses are the costs incurred by patients' inability to engage in economic activities during illness and treatment, and the total amount was calculated by multiplying the number of visits by the average daily income of the corresponding age group. For inpatient care, a hospital visit was counted as one day, while, for outpatient care, one visit was counted as one-third of a day. Those aged 20–64 were assumed to be the economically active population, while those in the other groups were not considered to incur CDI-associated economic losses.

Productivity loss arising from CDI-associated premature death was estimated by calculating each deceased person's income based on his/her salary level that year. Assuming that a person stops earning an income at the age of 65, we calculated the average annual income for each deceased person from the year of their death to the year they would have reached the age of 64.

Finally, caregiver support of CDI patients is an economic loss, as the caregiver may cease or decrease participation in economic activities for a set period. Assuming that the caregiver is female of age 50–54 years, we calculated the cost by multiplying the number of visits by the average daily income of the corresponding age and gender group [25]. Caregiver cost was applied to all inpatients regardless of age and to outpatients in the ≤19 and ≥65 age groups, as it was assumed that patients aged 20–64 did not require a caregiver [25, 26]. Information on average incomes was obtained from Korean Employment and Labor statistics [27]. We calculated the per capita cost in order to determine if the increased CDI-associated expenses were due to higher prevalence or higher severity. Cost per capita was calculated by dividing total cost by the number of patients. Statistical analyses were performed using SAS version 9.3 (SAS Institute, Cary, NC).

## 3. Results

### 3.1. Prevalence and Mortality

The number of CDI patients increased from 700 in 2008 to 1,177 in 2009, 1,714 in 2010, and 2,521 in 2011. The overall proportion of male patients was 36%. The proportion of those aged ≥65 years was 61.7% in 2008, 59.5% in 2009, 66.3% in 2010, and 67.0% in 2011. The number of CDI-associated deaths increased by 2.5-fold in three years, from 69 in 2008 to 172 in 2011. Female patients accounted for 62% of CDI-caused mortality (Table 1).

**Figure 1:**
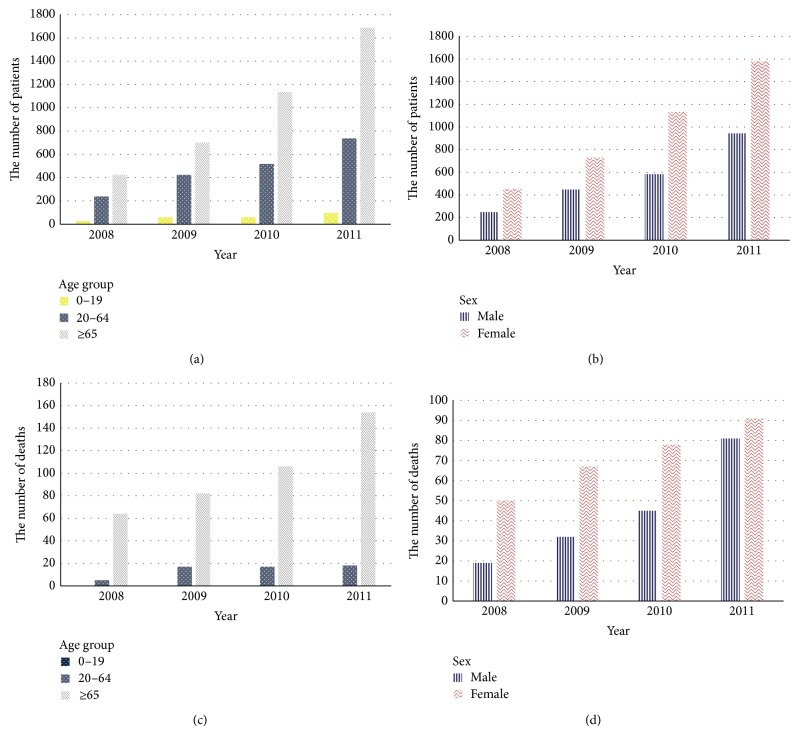
Four-year trend of* Clostridium difficile* infection according to subgroup. The number of patients with* Clostridium difficile* infection (a) and associated mortality (c) by age group. The number of patients with* Clostridium difficile* infection (b) and mortality (d) by gender.

For a more detailed analysis of the mortality trends, we separated fatal cases into five-year age groups and examined mortality rates. In 2008, mortality occurred only among individuals aged 60 years or older; however, five deaths occurred in the 45–49 age group in 2009 and the 35–39 age group saw one death in 2010 and two in 2011, illustrating that mortality now occurs among younger CDI patients. For a more accurate yearly comparison, we determined the prevalence and mortality per 100,000 persons. The prevalence increased steeply from 1.43 in 2008 to 2.39 in 2009, 3.47 in 2010, and 5.06 in 2011. Mortality also increased from 0.14 in 2008 to 0.20 in 2009, 0.25 in 2010, and 0.35 in 2011. The prevalence increased in all age groups and was especially marked in the ≥65 age group. Mortality increased every year for all groups, except for the 0–19 age group in which mortality did not occur and in 20–64-year-old women (Table 1, Figure 1).

### 3.2. Economic Burden

Using 2008 as the benchmark, the economic burden—the total cost obtained by summing the direct and indirect costs—increased from $2.4 million in 2008 to $7.6 million (3.12-fold) in 2009, $10.5 million (4.28-fold) in 2010, and $15.8 million (6.45-fold) in 2011 (Table 2). The rate of increase from the previous year's cost was 3.12-fold in 2009 and 1.37-fold in 2010. Direct and indirect costs increased each year compared to the 2008 figures (2009, 1.84-fold and 5.36-fold, resp.; 2010, 2.94-fold and 6.63-fold, resp.; 2011, 4.07-fold and 10.61-fold, resp.). This indicates that while both costs are increasing, the rate of increase was greater for the indirect costs than for direct costs. The per capita total cost increased by 2.09-fold in 2009, 2.12-fold in 2010, and 2.42-fold in 2011, compared to 2008, showing that the per capita total cost did not change markedly since 2009 (Table 2). When the total cost was compared between the male and female patients in each age group, the largest group was in 2008 in the ≥65 age group. In 2010 and 2011, however, the total cost was highest in the 20–64 age group for men and in the ≥65 age group for women. The per capita total cost was highest in the 20–64 age group, the economically active population, in every year (Table 2).

**Figure 2:**
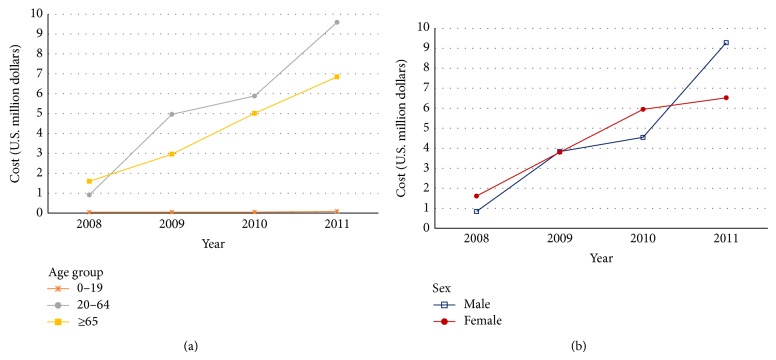
Four-year trend of total costs of* Clostridium difficile* infection by age group (a) and gender (b).

The yearly change in economic cost was 5.41-fold in 2009, 1.19-fold in 2010, and 1.62-fold in 2011 for the 20–64 years group, while the ≥65 age group showed a steady increase from one year to the next. By gender, the total costs for men were $0.8, $3.8, $4.5, and $9.3 million in 2008, 2009, 2010, and 2011, respectively, and those for women were $1.6, $3.8, $5.90, and $6.5 million, respectively. The total cost was higher for women until 2010, when the cost of CDI in men increased rapidly, far exceeding the cost for women in 2011 (Figure 2).

## 4. Discussion

*C. difficile *is one of the major causes of infectious diarrhea in hospitalized patients [6]. This study counted the number of CDI patients and deaths using nationwide data for Korea during the period of 2008–2011 and estimated the socioeconomic cost of CDI in Korea. This was the first study to examine CDI prevalence and mortality, as well as estimating the disease burden in Korea. In the three years following 2008, the number of CDI patients increased by 3.6-fold and the number of deaths by 2.5-fold; as a result, the socioeconomic cost rose by 6.45-fold during the study period. After 2009, the cost increased sharply due mainly to the marked rise in indirect costs.

Recently, the increase in total CDI-associated cost has been remarkable. Particularly, the total cost of 20–64 age male group and ≥65 age female group increased. These sharp increases could be supporting the other Korean study based on a single-hospital observation during September 2008 to January 2010 [32]. The incidence of CDI has increased since November 2009 and it peaked in December 2009 and our study has showed steady increase since 2009. These results support that the cases of CDI including mortality amplified these times and are similar to the result of Canada and USA with the epidemic of virulent strain [21].

**Figure 3:**
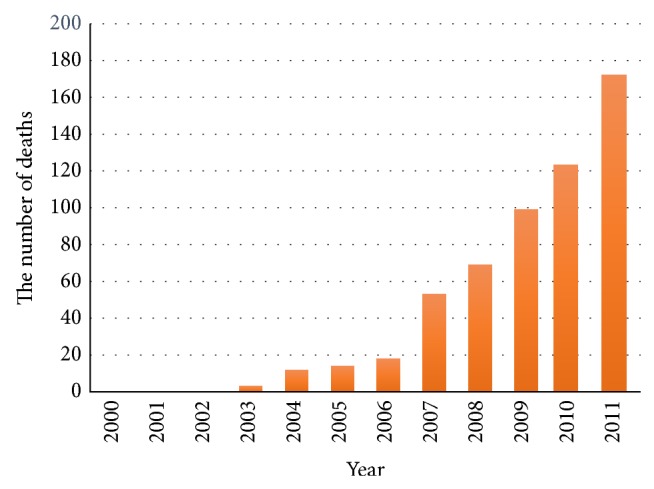
Twelve-year trend of the number of deaths caused by* Clostridium difficile* infection.

Along with this increase in overall cost, per capita cost rose as well, suggesting that worsened severity including death made a considerable contribution in addition to the increase in the number of patients; patients aged 20–64 especially had a significant increase in per capita total cost. This indicates the burden of each patient is most severe in 20–64 age group, while the overall economic burden could be larger in older age group. In particular, the increase each year in mortality among younger patients is responsible for heightened indirect cost. Regarding the long-term mortality trend during the period of 2000–2011, there was no CDI-associated mortality until 2002, while the number of deaths rose to three in 2003, 53 in 2007, and 172 in 2011 (Figure 3).

The use of antibiotics is a risk factor for CDI. In Korea, the use of antibiotics has increased from 24.28 defined daily doses (DDD) per 1000 person-days in 2008 to 25.17 DDD per 1000 person-days in 2009 [33]. Similarly, the economic value of Korea's antibiotic production market increased continuously from just over $127 million in 2008 to $130.7 million in 2009 and approximately $142 million in 2010, with a decrease to $134.7 million in 2011 [34]. In particular, the use of cephalosporins, a class of antibiotics strongly associated with CDI, has increased steadily since 2003 [32]. This trend is believed to also be related to the increasing number of CDI patients. The increase in CDI in Korea has been reported in previous studies. In a single-hospital study, the incidence of CDI increased 6-fold over 1998–2007, from 1.9 inpatients per 10,000 persons in 1998 to 8.82 in 2006-2007 [4]. In another study of 17 hospitals over the period of 2004–2008, the number of adult (aged over 19) inpatients per 1,000 persons increased from 1.7 in 2004 to 2.7 in 2008 [5]. Our study shows that this increase in Korea has continued during the period of 2008–2011.

In the USA, the estimated number of CDI patients doubled from 2001 to 2005, and 450,000–750,000 new cases were expected in 2010 [35]. The estimated economic cost was $13,310–$16,464 per CDI patient in 2008, bringing the total social cost to $796 million. These data show that CDI causes a huge burden of disease in the USA [35]. A study conducted in a country other than the USA reported similar results, estimating that additional per patient medical expenses caused by CDI were $5,243–$8,570 for first-onset cases and $13,655 for recurrent cases [6]. In Canada, the economic cost of CDI per year per facility is estimated as $117,712 [10]. Therefore, our study shows that the situation in Korea is consistent with the rapidly increasing CDI burden observed in other countries.

Mortality attributed to CDI has increased, particularly among young people, for whom the absence of underlying disease previously classified them as a low-risk group; this is similar to recent trends seen in other countries [36]. Further research should examine other factors, such as the patients' immune status or previous use of antimicrobials and the effect of ribotype in Korean CDI cases, among others.

While the 2008 social cost was $796 million in the USA, the economic burden was only $2.4–$15.8 million in Korea. This might be due in part to the difference in medical expenses between the two countries, in addition to a difference in analysis methods. By contrast, the percapita cost in Korea was $3,000–$7,000, which is lower than that of the USA but still relatively high. The economic cost of CDI varies widely. For example, per case cost of CDI was estimated at $6,689 in 1996 in the UK and at $4,782 in 2002 in Northern Ireland [8]. This is likely attributable to the data characteristics; the subjects included in this study were patients for whom CDI was the principal diagnosis, and their characteristics might differ from patients suspected of having CDI but who were not diagnosed. In the Korean National Health Insurance system, the disease with the largest treatment cost is regarded as the principal diagnosis, and so most of the cases included in this study were patients with severe CDI; as a result, the medical expense per capita was relatively high. In addition, this study showed that the burden of disease caused by CDI is rapidly increasing.

In comparison, estimated socioeconomic costs for acute coronary syndrome, asthma, and cancer were $864.8 million (2008), $831.1 million (2008), and $14 billion (2005), respectively [37, 38]. The estimated economic cost of CDI from this study was $2.4–$15.8 million, relatively, lower than that of other reported diseases, but the per capita cost of $6,554 is considerably higher than that of asthma ($366), suggesting that the socioeconomic burden of CDI is significant to individual patients [25].

Since the socioeconomic burden of CDI in Korea is rapidly increasing, it is important to consider a multifaceted approach to ameliorate this situation. Improvement of healthcare workers' hand hygiene and the implementation of a practical antimicrobial stewardship program based on local epidemiology may be useful [21]. For example, glycopeptide use is one of the risk factors for binary toxin-producing CDI in Korea [39]. Thus, limiting the number of patients being administered this type of antimicrobial therapy, as well as its duration, could reduce CDI risk.

This study had several limitations. Since it included cases where CDI was the principal diagnosis, the CDI patients analyzed might include only those who incurred the highest cost for CDI treatment, and consequently the overall cost might be underestimated. Conversely, while previous studies examining the CDI burden focused solely on inpatients, ignoring outpatients' costs, this study included outpatients who visited the hospital for CDI recurrence or for other reasons [40]. Moreover, although many studies have used health insurance data, the accuracy of the data is questionable because it comes from insurance claims [41]. The uninsured medical fees were estimated based on the percentage of benefits in the total amount of medical charges at a limited number of medical institutions only. This might deviate from the actual ratio of benefits to uninsured benefits and constitute a study limitation [30].

This study examined data over four years without showing a long-term trend due to limited available health insurance data. Nevertheless, despite this shortcoming, the burden of CDI from Korean source data shows a recent rapid increase in the prevalence and mortality associated with CDI. The cause of this trend must be identified, and effective interventions developed. CDI control is vital, since it is generally a nosocomial infection, which is preventable.

## Figures and Tables

**Table 1:** Four-year trend in *Clostridium difficile* infection.

Year	Sex	Age group	Number of patients	Number of deaths	Prevalence (per 100,000)	Mortality rate (per 100,000)
2008	M	0–19	20	0	0.32	0.00
		20–64	88	1	0.54	0.01
		≥65	140	18	6.85	0.88
	F	0–19	10	0	0.18	0.00
		20–64	150	4	0.95	0.03
		≥65	292	46	9.71	1.53
	Subtotal		**700**	**69**	**1.43**	**0.14**

2009	M	0–19	27	0	0.44	0.00
		20–64	166	7	1.01	0.04
		≥65	254	25	11.89	1.17
	F	0–19	26	0	0.47	0.00
		20–64	258	10	1.63	0.06
		≥65	446	57	14.30	1.83
	Subtotal		**1177**	**99**	**2.39**	**0.20**

2010	M	0–19	32	0	0.53	0.00
		20–64	189	9	1.14	0.05
		≥65	362	36	16.25	1.62
	F	0–19	28	0	0.51	0.00
		20–64	329	8	2.06	0.05
		≥65	774	70	24.00	2.17
	Subtotal		**1714**	**123**	**3.47**	**0.25**

2011	M	0–19	52	0	0.88	0.00
		20–64	289	12	1.73	0.07
		≥65	602	69	25.94	2.97
	F	0–19	44	0	0.82	0.00
		20–64	448	6	2.78	0.04
		≥65	1086	85	32.56	2.55
	Subtotal		**2521**	**172**	**5.06**	**0.35**

**Table 2:** Economic burden of *Clostridium difficile* infection^*^.

Year	Sex	Age group	Number of outpatient visits	Numbers of admission days	Direct cost	Indirect cost	Total cost	Per capita
2008	M	0–19	14	291	12	17	29	1.43
		20–64	76	726	153	191	343	5.05
		≥65	83	1,901	357	111	468	2.92
	F	0–19	5	64	9	4	13	1.28
		20–64	120	1,085	218	318	536	4.47
		≥65	213	4,300	808	251	1,059	3.29
	Subtotal		**511 **	**8,367 **	**1,556 **	**891**	**2,447 **	**3.07**

2009	M	0–19	15	71	14	4	18	0.66
		20–64	158	1,765	262	2,412	2,674	19.38
		≥65	158	4,062	908	235	1,143	4.05
	F	0–19	17	128	21	8	29	1.10
		20–64	236	1,722	357	1,729	2,086	9.93
		≥65	345	6,666	1,297	388	1,685	3.41
	Subtotal		**929 **	**14,414 **	**2,858 **	**4,776 **	**7,634 **	**6.42 **

2010	M	0–19	24	118	18	7	26	0.80
		20–64	171	1,638	329	2,742	3,071	20.07
		≥65	240	5,393	1,129	318	1,447	3.63
	F	0–19	30	91	18	6	24	0.85
		20–64	206	1,520	494	2,082	2,576	9.72
		≥65	585	13,624	2,591	751	3,342	3.99
	Subtotal		**1,256 **	**22,384 **	**4,579 **	**5,906 **	**10,485 **	**6.51 **

2011	M	0–19	50	129	34	9	43	0.83
		20–64	237	1,472	567	6,184	6,751	29.23
		≥65	479	9,680	1,951	531	2,482	3.76
	F	0–19	47	143	27	9	36	0.82
		20–64	336	2,007	636	1,788	2,424	6.55
		≥65	850	15,476	3,125	936	4,062	3.49
	Subtotal		**1,999 **	**28,907 **	**6,340 **	**9,457 **	**15,797 **	**7.45 **

^*^In 1,000 dollars.
